# Stepwise Conformational Stabilization of a HIV-1 Clade C Consensus Envelope Trimer Immunogen Impacts the Profile of Vaccine-Induced Antibody Responses

**DOI:** 10.3390/vaccines9070750

**Published:** 2021-07-06

**Authors:** Alexandra Hauser, George Carnell, Kathrin Held, Guidenn Sulbaran, Nadine Tischbierek, Lisa Rogers, Georgios Pollakis, Paul Tonks, Michael Hoelscher, Song Ding, Rogier W. Sanders, Christof Geldmacher, Quentin Sattentau, Winfried Weissenhorn, Jonathan L. Heeney, David Peterhoff, Ralf Wagner

**Affiliations:** 1 Institute of Medical Microbiology and Hygiene, Molecular Microbiology (Virology), University of Regensburg, 93053 Regensburg, Germany; Alexandra.Hauser@ukr.de (A.H.); Nadine1.Tischbierek@stud.uni-regensburg.de (N.T.); David.Peterhoff@ukr.de (D.P.); 2 Laboratory of Viral Zoonotics, Department of Veterinary Medicine, University of Cambridge, Madingley Road, Cambridge CB3 0ES, UK; gwc26@cam.ac.uk (G.C.); pt10001@cam.ac.uk (P.T.); jlh66@cam.ac.uk (J.L.H.); 3 Division of Infectious Diseases and Tropical Medicine, University Hospital, LMU Munich, 80802 Munich, Germany; kathrin.held@med.uni-muenchen.de (K.H.); rogers@lrz.uni-muenchen.de (L.R.); hoelscher@lrz.uni-muenchen.de (M.H.); geldmacher@lrz.uni-muenchen.de (C.G.); 4 German Center for Infection Research (DZIF), Partner site Munich, 80802 Munich, Germany; 5 Institut de Biologie Structurale (IBS), University Grenoble Alpes, CEA, CNRS, 38000 Grenoble, France; Guidenn.sulbaran-Machado@ibs.fr (G.S.); Winfried.Weissenhorn@ibs.fr (W.W.); 6 Department of Clinical Infection, Microbiology, and Immunology (CIMI), University of Liverpool, Liverpool L69 7BE, UK; G.Pollakis@liverpool.ac.uk; 7 NIHR Health Protection Research Unit in Emerging and Zoonotic Infections (HPRU EZI), Liverpool L69 7BE, UK; 8 EuroVacc Foundation, 1105 BP Amsterdam, The Netherlands; song.ding@eurovacc.org; 9 Department of Medical Microbiology, Academic Medical Center, University of Amsterdam, 1105 AZ Amsterdam, The Netherlands; r.w.sanders@amc.uva.nl; 10 Department of Microbiology and Immunology, Weill Medical College of Cornell University, New York, NY 10021, USA; 11 The Sir Willian Dunn School of Pathology, The University of Oxford, Oxford OX1 3RE, UK; quentin.sattentau@path.ox.ac.uk; 12 Institute of Clinical Microbiology and Hygiene, University Hospital Regensburg, 93053 Regensburg, Germany

**Keywords:** HIV vaccine, envelope, stabilized trimer, clade C, consensus, centralized sequence, immunization, immunogen design, antibody response, peptide microarray

## Abstract

Stabilization of the HIV-1 Envelope glycoprotein trimer (Env) in its native pre-fusion closed conformation is regarded as one of several requirements for the induction of neutralizing antibody (nAb) responses, which, in turn, will most likely be a prerequisite for the development of an efficacious preventive vaccine. Here, we systematically analyzed how the stepwise stabilization of a clade C consensus (ConC) Env immunogen impacts biochemical and biophysical protein traits such as antigenicity, thermal stability, structural integrity, and particle size distribution. The increasing degree of conformational rigidification positively correlates with favorable protein characteristics, leading to optimized homogeneity of the protein preparations, increased thermal stability, and an overall favorable binding profile of structure-dependent broadly neutralizing antibodies (bnAbs) and non-neutralizing antibodies (non-nAbs). We confirmed that increasing the structural integrity and stability of the Env trimers positively correlates with the quality of induced antibody responses by the immunogens. These and other data contribute to the selection of ConCv5 KIKO as novel Env immunogens for use within the European Union’s H2020 Research Consortium EHVA (European HIV Alliance) for further preclinical analysis and phase 1 clinical development.

## 1. Introduction

The HIV-1 Envelope surface glycoprotein (Env) is a trimer of heterodimers composed of non-covalently associated gp120 and gp41 subunits, which mature from a gp160 precursor unpon enzymatic cleavage by cellular proteases. Env mediates binding to the CD4 receptor on immune cells and, upon a series of conformational changes, facilitates infection (as reviewed by [1]). Antibodies targeting Env in its native conformation can prevent binding to the CD4 receptor or the CCR5 or CXCR4 co-receptor on target cells, or interfere with the fusion process, and thereby prevent infection. However, the induction of neutralizing antibodies (nAbs) is hampered by the dense glycan shield on Env, which covers almost the entire protein surface and thereby restricts accessibility for antibodies [2]. This, together with the structural flexibility and high degree of sequence variability of Env (up to 35% across all group M clades [3]) are regarded as the main impediments to the design of a successful vaccine capable of inducing antibodies that can neutralize a broad variety of HIV-1 strains (broadly neutralizing antibodies, bnAbs) (as reviewed by [4,5] and others). Therefore, Env immunogen candidates that are stabilized in a native, pre-fusion closed conformation are desirable, and centralized immunogen sequences [3,6] that represent circulating virus isolates as closely as possible would be additionally beneficial for the development of breadth.

Over the past two decades, HIV-1 Env trimer-based immunogens have undergone fundamental improvements. The non-covalent association of the gp120 and gp41 subunits, which lead to gp120 dissociation and shedding of gp120, particularly on soluble forms of Env, was overcome by two strategies: (i) The introduction of a disulfide bridge between gp120 and gp41 (A501C/T605C) in combination with the I559P substitution in gp41 prevented shedding of gp120 and stabilized the trimer [7,8]. (ii) The replacement of the furin cleavage site by a flexible linker of suitable length allowed cleavage-independent trimers [9]. These strategies contributed to the generation of closed, native-like trimers with reduced exposure of epitopes that induce non-neutralizing antibodies [10]. This is of particular importance, as B-cell responses to off-target immunodominant regions such as V3 (as reviewed by [11]) might compete with other subdominant B-cells, recognizing weakly immunogenic neutralization-relevant regions in the germinal centers [12]. Today, a variety of native-like trimers from different viral clades are available [13,14,15,16,17] and continuous optimization of stabilized Env trimers for favorable immunological traits is ongoing (as reviewed by [18]). These immunogens, however, were derived from distinct viral isolates and are therefore likely to induce strain-specific responses with low cross-reactivity.

Induction of more broadly reactive antibody responses might be promoted by genetically and antigenically ‘centralized’ immunogens such as consensus, ancestor, or mosaic proteins, which aim at representing the diversity of circulating viruses. Mosaic immunogens, being enriched for T-cell or B-cell epitopes, have demonstrated their ability to induce improved cellular immune responses [19]. While ancestral sequence reconstruction infers phylogenetic relationships, the generation of a consensus sequence utilizes the most frequently observed amino acids in each position of a multiple sequence alignment and therefore provides a sequence most closely related to all input sequences [20]. Notably, an ancestral clade C SOSIP gp140 trimer has proven successful to induce broadly neutralizing nanobodies against HIV-1 in camels [21]. Currently, only a small number of native-like Env trimer constructs derived from consensus sequences are being explored. A soluble stabilized Env trimer was generated with a consensus sequence of clade C [22] and the underlying repair-and-stabilization concept (RnS) was successfully transferred to representative isolates from other clades [23]. Neither study, however, assessed the immunogenicity of these trimers. Further, the Env trimer ConM SOSIP, based on a consensus sequence covering the complete group M, induced neutralizing antibody responses in rabbits and macaques [24], and is currently being tested in a clinical trial in humans (NCT03816137) [25].

The only phase 3 clinical trial so far to show moderate efficacy of 31% in preventing HIV-1 infection after 3 years was the RV144 trial in Thailand [26]. Several immunological correlates of protection were identified [27], which include not only neutralizing antibodies but for example also binding antibodies targeting the V1V2 region in Env. The protective effects of RV144, however, could so far be transferred neither to other populations nor to different immunogens. Therefore, there is still an urgent need to explore new potential immunogens and immunization schedules.

Here, we present a set of Env variants derived from a novel clade C consensus sequence (ConC). Viruses from HIV-1 clade C account for almost one half of global infections and are furthermore overrepresented in the high prevalence areas of sub-Saharan Africa and south-east Asia [28]. Regrettably, sub-optimal ART coverage is also reported for these regions [29]. The panel of Env proteins we have generated consists of one gp120 monomer and four trimers that were generated by stepwise inclusion of amino acid substitutions with known impact on the protein’s propensity to form pre-fusion closed trimers. On top, selected N-linked glycan sites were modified in order to facilitate binding of bnAb 2G12 for affinity purification (KI; glycan knock-in) [30,31] and in order to allow for improved accessibility of the CD 4bindig site (KO; glycan knock-out) [32]. We systematically analyzed the positive impact of the stepwise modification of ConC KIKO Env variants on biochemical and biophysical protein traits and on antigenicity using a panel of well-characterized antibodies targeting the major sites of vulnerability of Env. In addition, we demonstrate that the favorable in vitro characteristics translate into a beneficial immunological outcome in immunized Balb/c mice.

## 2. Materials and Methods

### 2.1. Construct Design

The HIV-1 clade C consensus sequence (ConC) was derived from a multiple-sequence alignment from all available sequences from Los Alamos Sequence Database (cut-off date: 1 April 2016), with restriction to one sequence per patient, using the default method of the platform’s Search Interface. The gp120 monomer and the gp140 trimer constructs were truncated at HXB2 (GeneBank Accession: K03455) positions 507 and 664, respectively, and the autologous signal peptide was replaced by the signal peptide from the tPA (tissue plasminogen activator) serine protease [33]. The N-linked glycan at position 295 (V295N substation) was introduced to restore binding to antibody 2G12 [30,31], and the N-linked glycans in positions 276 (N276Q substitution) and 462/463 (tandem sequon, N463Q/T464A substitutions) were removed by substitution of asparagine to glutamine for better accessibility of the CD4 binding site (CD4bs) in an immune focusing approach [32]. The resulting constructs were termed KIKO (KI: knock-in of 2G12 relevant N-linked glycans; KO: knock-out of N-linked glycans around the CD4bs). The different ConC trimer versions ConCv1, v2, v4, and v5 were generated essentially as follows: Briefly, for ConCv1 KIKO NFL, only the cleavage site REKR was replaced by (G_4_S)_2_, a glycine-serine based native flexible linker (NFL), in order to obtain cleavage independent trimers where the gp120 and gp41 subunits were covalently linked [9]. For ConCv2 KIKO, the SOSIP mutations consisting of a disulfide bridge A501C/T605C between gp120 and gp41 and an I559P substitution in gp41 (for pre-fusion state stabilization) were included [7,8]. In addition, for ConCv4 KIKO, a set of eight amino acid substitutions (E47D, K49E, V65K, E106T, I165L, E429R, R432Q, and K500R) termed TD8 [34] and corresponding amino acid substitutions in spatial proximity (unpublished) were introduced. Furthermore, for ConCv5 KIKO, an additional group of six amino acid substitutions for inhibition of transition to the CD4i state (H66R/T316W) and for enhanced trimer formation (I535M/Q543N) [35] as well as for additional covalent linkage between gp120 and gp41 (disulfide bond A73C-A561C) [36] were introduced.

### 2.2. Expression and Purification of Recombinant Env Proteins

Plasmids encoding for the *env* genes of Env variants AMC008 SOSIPv4.2 and AMC011 SOSIPv4.2 were kindly provided by Rogier W. Sanders (AMC, Amsterdam, The Netherlands). All used Envelope variants had a C-terminal hexa-histidine tag (His6) for purification that was directly fused to the Env proteins via a glycine-serine-linker (GS). Briefly, FreeStyle™ 293-F cells (Thermo Fisher Scientific, Waltham, MA, USA) cells were cultivated in FreeStyle™ 293 Expression Medium (Thermo Fisher Scientific, Waltham, MA, USA) and transfected with 1 µg DNA/mL at a cell density of 1 × 10^6^ cells/mL with plasmids encoding for the *env* and *furin protease* genes at a 1:3 ratio using polyethylenimine (PEI; Polysciences Inc., Warrington, PA, USA). In the case of variants that did not require cleavage by the furin protease (ConC KIKO gp120, ConCv1 KIKO NFL and 96ZM651 REKS.664), only plasmid DNA encoding for the respective *env* genes was used. Supernatants were harvested 5 days after transfection. Env proteins were purified by immobilized metal ion affinity chromatography (IMAC) using HisTrap excel columns (Cytiva, Marlborough, MA, USA) according to the manufacturer’s recommendations followed by buffer exchange to PBS. Proteins were subsequently further purified to homogeneity by size exclusion chromatography (SEC) using a Superdex^®^ 200 Increase 10/300 GL Column (Cytiva, Marlborough, MA, USA). The fractions representing the main peak were analyzed by BN PAGE (Blue Native Polyacrylamide Gel Electrophoresis). Suitable fractions were pooled for further use. Analytical SEC runs were performed with 10 µg of protein being loaded on a Superdex^®^ 200 Increase 10/300 GL Column (Cytiva, Marlborough, MA, USA) at a concentration of 0.1 µg/mL via a 100 µL sample loop overloaded with a suitable volume.

### 2.3. Monoclonal Antibodies

Antibodies 5F3 and 2G12 were purchased from Polymun Scientific, Austria. Antibodies and plasmids for antibodies PGT145 and PGT151 were kindly provided by Dennis Burton (The Scripps Research Institute, Torrey Pines Rd, La Jolla, CA, USA). Antibody and plasmids for antibody 10-1074 were kindly provided by Michel Nussenzweig (The Rockefeller University, New York, NY, USA). Plasmids for antibodies 14E and 19b were kindly provided by James Robinson (CHAVI-ID, USA). The following reagents were obtained through the NIH HIV Reagent Program, Division of AIDS, NIAID, NIH: Anti-Human Immunodeficiency Virus 1 gp120 Monoclonal Antibody (PG9), ARP-11557; Anti-Human Immunodeficiency Virus (HIV)-1 gp120 Monoclonal Antibody (PG16), ARP-12150; and Anti-Human Immunodeficiency Virus (HIV)-1 gp120 Monoclonal Antibody (PGT121), ARP-12343, were contributed by International AIDS Vaccine Initiative. Human Immunodeficiency Virus 1 (HIV-1) VRC01 Monoclonal Antibody Heavy Chain Expression Vector, ARP-12035 and Human Immunodeficiency Virus 1 (HIV-1) VRC01 Monoclonal Antibody Light Chain Expression Vector, ARP-12036, were contributed by John Mascola. Anti-Human Immunodeficiency Virus (HIV)-1 gp120 Monoclonal Antibody (F105), ARP-857, was contributed by Dr. Marshall Posner and Dr. Lisa Cavacini. Human Immunodeficiency Virus 1 (HIV-1) 447-52D mAb Heavy Chain Expression Vector, ARP-13617 and Human Immunodeficiency Virus 1 (HIV-1) 447-52D mAb Light Chain Expression Vector, ARP-13618 were contributed by Dr. Susan Zolla-Pazner. Monoclonal Anti-Human Immunodeficiency Virus Type 1 (HIV-1) gp120 Protein, Clone 17b (produced in vitro), ARP-4091, was contributed by Dr. James E. Robinson; Plasmid pcDNA3.1(-) Expressing Human CD4 Fused with Human IgG1 Hinge and Fc Regions, ARP-12960, was contributed by Dr. Xueling Wu. For in-house production of monoclonal human anti-Env antibodies, plasmids encoding for the respective heavy and light chain genes were transfected to either FreeStyle™ 293-F cells or Expi293F™ cells (Thermo Fisher Scientific, Waltham, MA, USA) at a 1:1 ratio. Transfection of FreeStyle™ 293-F cells was performed essentially as described above. Expi293F™ cells were cultivated in Expi293™ Expression Medium (Thermo Fisher Scientific, Waltham, MA, USA) and transfected at a density of 2.9 × 10^6^ cells/mL using the ExpiFectamine™ 293 Transfection Kit (Thermo Fisher Scientific, Waltham, MA, USA) according to the manufacturer’s recommendations. In both cases, supernatants were harvested 5 days post transfection and purified by Protein A affinity chromatography using HiTrap rProtein A FF columns (Cytiva, Marlborough, MA, USA).

### 2.4. Blue Native PAGE

Blue Native PAGE (BN PAGE) was performed for verification of the purity of the trimer pools. Therefore, 2 µg protein per lane was loaded on a SERVAGel™ N 4–16 (SERVA Electrophoresis GmbH, Heidelberg, Germany) and run for 20 min at 50 V followed by 4 h at 100 V. Gels were stained for 10–20 min with Coomassie staining solution (1.25% (*w*/*v*) Coomassie Brilliant Blue R-250, 50% (*v*/*v*) ethanol, 7% (*v*/*v*) glacial acetic acid) and destained with 7% (*v*/*v*) acetic acid overnight.

### 2.5. SDS PAGE

Reducing and non-reducing SDS-PAGEs were performed for verification of complete cleavage of the gp120 and gp41 subunits (where applicable). Briefly, 2 µg of protein were denatured for 5 min at 95 °C, samples were loaded on pre-cast SERVAGel™ TG PRiME™ 8–16% gels (SERVA Electrophoresis GmbH, Heidelberg, Germany) and run at constant voltage for 10 min at 100 V followed by 45 min at 250 V. Staining and destaining of the gels were performed as described for BN PAGEs.

### 2.6. Animal Study

Eight-week old Balb/c females were ordered from Charles River Laboratories, UK and housed at University Biomedical Services, University of Cambridge. Animal work was carried out under project license P8143424B with approval from the Animal Welfare Ethical Review Body, University of Cambridge.

Mice were immunized with 20 µg of Env trimers adjuvanted with monophosphoryl lipid A liposome solution (Polymun Scientific, Klosterneuburg, Austria), a Toll-like receptor 4 agonist, at a 1:2.5 ratio in eight groups. Immunizations were administered in 100 µL subcutaneously in the rear flank. Animals received a total of three immunizations at weeks 0, 4, and 8, and sera were collected at the pre-immune state and 2 weeks after each immunization (weeks 0, 2, 6, and 10). Animals were terminally bled at week 10 by cardiac puncture under non-recovery anesthesia.

### 2.7. Enzyme-Linked Immunosorbent Assay (ELISA)

Binding affinities of monoclonal antibodies to various Env trimer variants were determined by Ni-NTA capture ELISA. Briefly, 350 ng Env gp140 trimer or the triple molar amount of gp120 monomer (261 ng) was coated onto Ni-NTA HisSorb plates (Qiagen, Hilden, Germany) over night at 4 °C. Plates were washed with TBS and serial dilutions of the monoclonal antibodies were added in PBS containing 2% skimmed milk and incubated for 2 h at room temperature. After washing with TBS, the detection antibody (rabbit anti-human IgG/HRP, P0214, Agilent, CA, USA) was added at a 1:5000 dilution in PBS containing 1% BSA and incubated for 1 h. After washing with TBS, plates were developed using in-house TMB substrate solution (1:20 dilution of substrate (1 mM TMB, 80 mM H_2_O_2_, 10% (*v*/*v*) acetone, 90% (*v*/*v*) ethanol) in dilution buffer (30 mM tripotassium citrate)) and the reaction was stopped with 1 M sulfuric acid. The absorbance was measured at 450 nm (specific signal) and 655 nm (background) on an iMark™ Microplate Reader (Biorad, Hercules, CA, USA). Data were analyzed using GraphPad Prism Software (GraphPad Software Inc., San Diego, CA, USA).

### 2.8. Env Binding IgG antibody Responses in Mouse Sera

Binding antibody titers in sera of mice immunized with different Env variants were determined by capture ELISA. Briefly, 350 ng of Env gp140 trimers or 261 ng of Env gp120 monomer were coated onto Ni-NTA HisSorb plates (Qiagen, Hilden, Germany) for 2 h at room temperature or overnight at 4 °C. Plates were washed with TBS and blocked for 30 min with PBS containing 0.1% (*w*/*v*) I-BlockTM (Invitrogen, Waltham, MA, USA) and 1x Roti^®^Block (Carl Roth, Karlsruhe, Germany) (blocking buffer). Plates were washed with TBS and serial dilutions of mouse sera starting at a 1:50 dilution were added in blocking buffer and incubated for 2 h at room temperature. After washing with TBS, the detection antibody (goat anti-mouse IgG/HRP; Jackson ImmunoResearch, West Grove, PA, USA) was added at a 1:10,000 dilution in PBS containing 1% (*w*/*v*) BSA and incubated for 1 h at room temperature. Plates were washed with TBS supplemented with 0.05% (*v*/*v*) Tween and again with TBS in order to remove residual Tween. Plates were developed as described for monoclonal antibodies. Midpoint titers were calculated using GraphPad Prism Software (GraphPad Software Inc, San Diego, CA, USA). Absence of Env reactivity in the pre-immune sera was confirmed for each animal towards the respective immunogen.

### 2.9. V1V2 Responses

The following reagents were obtained through the NIH HIV Reagent Program, Division of AIDS, NIAID, NIH: Human Immunodeficiency Virus 1 (HIV-1) Env V1-V2 Recombinant Protein (AE.A244 V1-V2.tags), ARP-12567 and (C.1086 V1-V2.tags), ARP-12568, contributed by Drs. Barton F. Haynes and Hua-Xin Liao. For analysis of V1V2 reactive antibodies, 70 ng of Env V1V2 recombinant protein was coated onto Ni-NTA HisSorb plates (Qiagen, Hilden, Germany) and ELISA analysis was performed as described for Env reactive antibody responses. AUC (area under curve) analysis was carried out using GraphPad Prism Software (GraphPad Software Inc., San Diego, CA, USA).

### 2.10. Luminex Binding Antibody Multiplex Assay

Breadth of serum reactivity was assessed towards a panel of Env variants including trimers from several viral clades (clade A: BG505 SOSIP.664, clade B: AMC008 SOSIPv4.2, AMC011 SOSIPv4.2, clade C: ConCv1 KIKO NFL, ConCv2 KIKO, ConCv4 KIKO, ConCv5 KIKO, 16055 SOSIP, 96ZM651 REKS.664) and one clade C gp120 monomer (ConC KIKO gp120). MagPlexAvidin Microspheres (2000 beads/test; Luminex, Austin, TX, USA) were washed with PBS, 2 mM EDTA, 1% BSA, 0.05% Tween-20, 0.1% ProClinTM 300 (Sigma Aldrich, St. Louis, MO, USA) (dilution buffer) and then coated at 250 beads/µL with in-house biotinylated anti-His6 IgY (Thermo Fisher Scientific, Waltham, MA, USA) at 5 µg/mL in dilution buffer for 1 h at RT. After washing with dilution buffer, the trimeric antigens were captured at 15 µg/mL and the monomeric gp120 was captured at an equimolar amount over night at 4 °C. After washing with dilution buffer, beads were further washed with PBS containing 0.1% (*w*/*v*) I-BlockTM (Invitrogen, Waltham, MA, USA) and 1x Roti^®^Block (Carl Roth, Karlsruhe, Germany) (blocking buffer) and then blocked for 1 h at RT. After blocking, the different bead regions were pooled and distributed in a 96-well plate (Greiner, Kremsmünster, Austria) and sera were applied in blocking buffer at a 1:80 dilution in a volume of 50 µL and incubated for 2 h at RT. After incubation, beads were washed with blocking buffer and again with dilution buffer. Then a R-Phycoerythrin-conjugated donkey anti-mouse IgG (H + L) AffiniPure F(ab’)2 fragment (Jackson ImmunoResearch, West Grove, PA, USA) was added at a 1:200 dilution for detection and incubated for 1 h at RT. After washing, beads were resuspended in dilution buffer and measurement was performed with a MAGPIX^®^ device (Luminex, Austin, TX, USA). A response was defined as being positive if the specific, background corrected, signal for an individual animal was higher than (1) two times the mean signal of non-immunized animals and (2) the mean plus three times the standard deviation of sera from non-immunized animals.

### 2.11. Nano Differential Scanning Fluorimetry (nanoDSF)

Thermostability of the trimers was characterized by thermal unfolding with a Prometheus NT.48 nanoDSF (NanoTemper Technologies GmbH, Munich, Germany). Proteins were diluted to a final concentration of 0.05 mg/mL. After loading the samples to High Sensitivity capillaries (NanoTemper Technologies GmbH, Munich Germany), intrinsic fluorescence was measured at a ramp rate of 1 °C/min with an excitation power of 30%. Protein unfolding was monitored by the changes in fluorescence emission at 350 and 330 nm. The thermal unfolding midpoint (Tm) of the proteins was determined with Prometheus NT software.

### 2.12. Dynamic Light Scattering (DLS)

Dynamic Light Scattering was used for isothermal particle sizing. Briefly, proteins were diluted to a final concentration of 2.5 µM and loaded to High Sensitivity capillaries (NanoTemper Technologies GmbH, Munich, Germany). Measurements were carried out at 20 °C in triplicates of 10 acquisitions each with a Prometheus Panta device (NanoTemper Technologies GmbH, Munich, Germany). The hydrodynamic radius of the particles in solvated state and the polydispersity index (PDI) of the samples were determined with the associated software.

### 2.13. Negative Staining Electron Microscopy and Image Processing

HIV Env proteins were visualized by negative-stain electron microscopy (EM) using 3–4 µL aliquots at concentrations of 0.1–0.2 mg/mL. Samples were applied for 10 s onto a mica carbon film and transferred to 400-mesh Cu grid that had been glow discharged at 20 mA for 30 s and then negatively stained with 2% (wt/vol) Sodium silicotungstate (SST) for 30 s. Previous to data collection, the grids were screened to assess stain quality and particle distribution. Data were collected on an FEI Tecnai T12 LaB6-EM operating at 120 kV accelerating voltage with a pixel size of 2.8 Å on the specimen plane using a Gatan Orius 1000 CCD Camera. On average, 30–40 micrographs were collected per sample. Classification of closed and open trimers was performed as described [16]. Briefly, two-dimensional (2D) class averaging was performed with the software Relion [37], using 35,829 particles for the analysis of ConCv2 KIKO; 56,407 particles for ConCv4 KIKO; and 55,248 particles for ConCv5 KIKO. The 2-D classes were then segregated into three structural groups, closed or open-native particles and non-native particles.

### 2.14. Peptide Microarray Design

Custom peptide microarrays were designed to systematically map and compare IgG recognition of linear HIV-1 Env regions in preclinical vaccine studies of the EHVA consortium and consisted of 15-mer peptides overlapping by 11 amino acids. Envelope sequences matching EHVA vaccine candidates were therefore included in the array design. These include: 96ZM651_AF286224, BG505_DQ208458, ConC, HKM3, ngp41CM, CN54gp140_AF286226, MVA-CMDR_AFJ93253, and unpublished. In addition to these, multiple other peptide variants were included for 15 previously identified immunodominant regions. These were identified in studies on different vaccine trials and natural HIV infection conducted by us and others [38,39,40,41,42,43]. Peptide variants covering these immunodominant regions in more depth with respect to the antigenic variation were selected as follows: All existing pre-seroconversion (n = 913) and recent (n = 723) HIV infection sequences from 192 subjects collected within 2010–2018 were obtained from the HIV database (www.hiv.lanl.gov (accessed on 29 May 2018)). These sequences were interrogated to identify the most frequently occurring molecular forms for the 15 selected Env regions. Moreover, peptide variants frequently identified in previous studies ([38,39,40,41,42,43] and unpublished data) are included to bridge newly generated data to results from these earlier studies. Peptides included in the microarray are representative of all HIV-1 clades, however, due to the focus on EHVA immunogens, Clade C is overrepresented. In total, 1034 overlapping 15-mer peptides cover the gp160 extracellular domains of the selected back-bone immunogen sequences and, in addition, frequently occurring antigenic variants within 15 selected immunodominant regions of specific interest. Each peptide is printed on the array in triplicates.

### 2.15. Peptide Microarray Mapping of HIV Env-Specific IgG Responses in Vaccinated Mice

Mouse sera were analyzed for the presence of HIV-1 Env-specific IgG antibodies as described before [40] with minor modifications. After blocking of the peptide microarray slides with blocking buffer (0.1% I-Block Protein-Based Blocking Reagent, Thermo Fisher Scientific, Waltham, MA, USA and 1 × ROTI Block, Carl Roth, Karlsruhe, Germany, in PBS), mouse sera were diluted 1:250 in blocking buffer and incubated for 2 h at RT. Bound mouse IgG antibodies were then detected using a cross-adsorbed goat anti-mouse IgG DyLight 650 antibody (Thermo Fisher Scientific, Waltham, MA, USA). Baseline (w0) and post-vaccination (w10) sera of each mouse were processed in the same run, with the baseline samples used for background determination. Microarrays were scanned using a GenePix 4000A scanner at 650 (signal) and 532 nm (background). Resulting images were analyzed using the GenePix Pro 6.0 software (Molecular Devices, San José, CA, USA) by overlaying the array layout encoded in an array-specific grid (gal file). Layout positioning was then controlled manually for accuracy. Results were exported from GenePix Pro 6.0 as gpr files, which link each position on the array with the measured fluorescence intensity (FI) of bound secondary antibody. These were processed using R to calculate the mean FI of the triplicate peptides, which excludes outliers. These resulting mean FI values, linked with each 15-mer peptide sequence, are then assigned to the alignment of all sequences included in the microarray contained in a single fasta file and base-line (w0) values are subtracted from post-vaccination (w10) values. FI values for each amino acid position in the Env alignment are then calculated from FI values of overlapping peptides. To calculate the frequency of responders (FOR) or mean FI per vaccination group, the variant with the strongest response for each position within the backbone alignment was used. IgG responses against individual amino acid positions were considered positive, if the corresponding mean FI value was above 200 after subtraction of the pre-vaccination value. Mean FI values of all mice in one group were calculated, if at least 2 out of the 6 mice showed a positive response against the corresponding array position. Immunodominant antigenic regions (IDRs) were defined as being recognized by at least 60% of mice per immunogen group at the post-vaccination time point and with a mean FI for individual amino acids above 10,512.17 (mean of all responses in all groups + 2 × SD). For variant comparisons, the mean response over all peptide variants starting at HXB2 Env position aa304 was calculated per vaccination group. Calculations were carried out using R version 3.5.1 and Microsoft Excel.

## 3. Results

**Figure 1:**
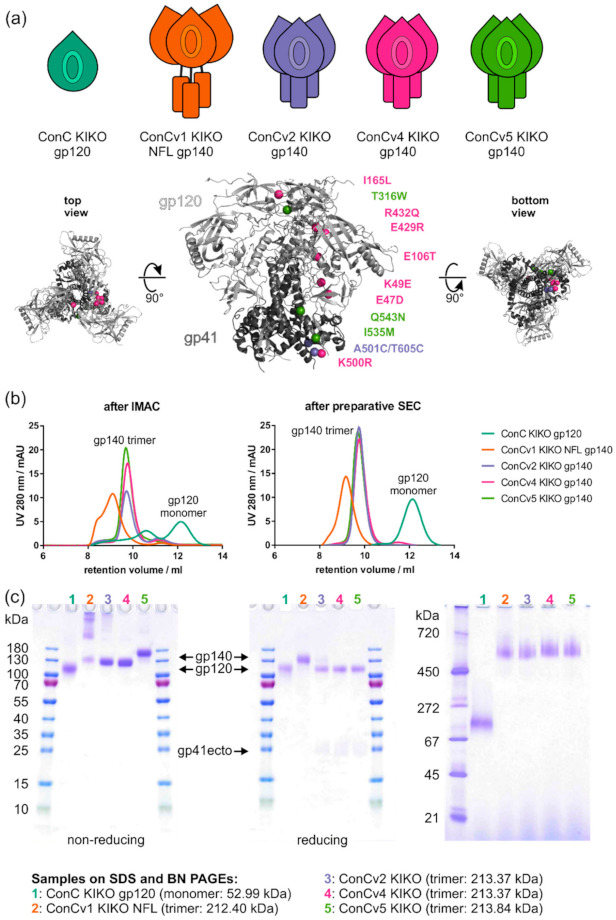
Purification of ConC Env variants for biophysical analyses and use as vaccines. (**a**) Schematic representation of the set of five ConC-derived proteins consisting of one gp120 monomer (ConC KIKO gp120, teal) and four gp140 trimers with stepwise increasing number of stabilizing modifications (ConCv1 KIKO NFL, orange; ConCv2 KIKO, lilac; ConCv4 KIKO, pink; ConCv5 KIKO, green). All included modifications are depicted and mapped on a pre-fusion Env trimer structure (PDB: 6CK9; ConC_Base0), as far as the respective regions were resolved. I559P first included in ConCv2 KIKO gp140; V65K first included in ConCv4 KIKO gp140; and H66R, A73C, and A561C first included in ConCv5 KIKO are hence not displayed. Colors are used according to the ConC Env version where the respective modification was first introduced. (**b**) Chromatograms of analytical size exclusion (SEC) runs with 10 µg of protein loaded directly after immobilized metal affinity chromatography (IMAC) (**left**) or after subsequent preparative SEC (**right**). (**c**) Non-reducing (**left**) and reducing (**middle**) SDS PAGEs for confirmation of correct cleavage and BN PAGE (**right**) for confirmation of purity of trimer in the final protein pool. Molecular weight for the gp120 monomer and the gp140 trimers are indicated, respectively. Two micrograms of protein were loaded per lane.

This study systematically assessed the impact of a stepwise stabilization of a soluble gp140 trimer by stepwise locking the Env trimers in the pre-fusion closed conformation determined based on their biochemical and biophysical characteristics. Furthermore, we were interested to analyze how potential biochemical and biophysical differences amongst the five Envs translated into distinct serum reactivity profiles in an inbred mouse model. We generated a gp120 monomer to mimic shedding of the non-covalently linked external glycoprotein moiety (ConC KIKO gp120) and next fused the gp120 and gp41 subunits in a cleavage-independent manner via a natural flexible linker [9] without any further modifications (ConCv1 KIKO NFL). Further, more advanced stabilized trimer variants were based on the cleavage dependent SOSIP modifications [7,8] alone (ConCv2 KIKO) or in combination. These include previously described modifications to improve trimer stability (ConCv4 KIKO) [34] and modifications that further increase trimerization and stability while preventing transition to the CD4-induced state (ConCv5 KIKO) [35,36] (Figure 1a).

### 3.1. Standardized Protein Purification Compensates for Inherent Differences in Native Protein Quality

All Env variants were purified by immobilized metal affinity chromatography (IMAC) by virtue of the hexa-his tag and subsequent size exclusion chromatography. To probe the native composition of Env protein species (monomer, dimer, trimer, aggregate) at the time of secretion from the cells, we performed analytical size exclusion chromatography (SEC) runs (Figure 1b left) directly after IMAC affinity purification without any further manipulation of the eluate that might affect protein quality. While the retention volumes of the more closed trimers ConCv2 KIKO, ConCv4 KIKO, and ConCv5 KIKO were fairly similar at 9.7 mL, the earlier elution of ConCv1 KIKO NFL at 9.1 mL suggested a more open conformation. Additional peaks or shoulders that eluted prior the trimer fractions represented aggregated protein (especially in ConCv1 KIKO NFL), while dimer or monomer eluted later (mainly visible for ConCv2 KIKO and ConCv4 KIKO). ConCv5 KIKO showed the most uniform peak and therefore appeared to primarily form trimers. The gp120 monomer eluted in two peaks that turned out to be dimerized or even multimerized gp120 subunits (peak at 10.6 mL with shoulder) and genuine gp120 monomers (peak at 12.1 mL).

To be able to analyze the properties of the predominant structural species, we chose to further purify them via SEC towards homogeneity. Blue native PAGEs of the protein pools after preparative SEC (Figure 1c right) and further analytical SEC runs of these pools (Figure 1b right) confirmed that protein species for the four gp140 trimers as well as for the gp120 monomer were largely homogeneous. Complete cleavage and absence of cleavage were confirmed for the SOSIP-based trimers v2/v4/v5 and the NFL-based trimer v1, respectively, by non-reducing and reducing SDS PAGEs (Figure 1c left, middle). Of note, the increasing degree of conformational rigidification positively correlated with trimer yields that could be purified from 1 L of transfected FreeStyle™ 293-F cells (ConCv1 KIKO NLF: 0.3 mg, ConCv2 KIKO: 0.6 mg, ConCv4 KIKO: 2.4 mg, ConCv5 KIKO: 4.5 mg, for comparison ConC KIKO gp120: 1.8 mg).

### 3.2. Increasing Degree of Stabilization Manifests in Biophysical Protein Properties

**Figure 2:**
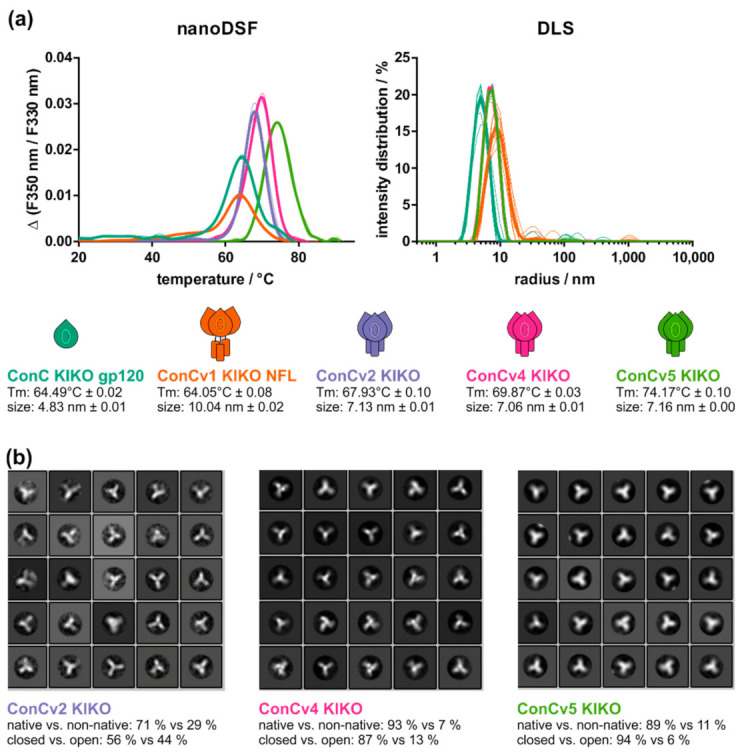
Stepwise stabilization leads to differences in biophysical protein traits. (**a**) nanoDSF and DLS analyses of all ConC-derived variants. Thermal transition curves (triplicates, mean in bold) are shown for one representative of three experiments. Intensity particle size distributions (10 acquisitions, mean in bold) are shown for one representative of three measurements. Melting temperatures (Tm) and particle sizes are indicated as mean ± SEM (standard error of mean) for each variant. ConC KIKO gp120: teal; ConCv1 KIKO NFL: orange; ConCv2 KIKO: lilac; ConCv4 KIKO: pink; ConCv5 KIKO: green. (**b**) Representative 2-D class averages of negative staining EM data of ConCv2 KIKO (**left**), ConCv4 KIKO (**center**), and ConCv5 KIKO (**right**). Indicated below each panel are the corresponding proportions of native vs. non-native trimers and closed vs. open trimers among native timers.

Thermostability and structural integrity were analyzed by nano-differential-scanning-fluorimetry (nanoDSF), negative staining electron microscopy (EM), and dynamic light scattering (DLS). Melting temperatures of the ConC KIKO gp120 monomer and the ConCv1 KIKO NFL timer, where gp120 and gp41 were only connected by a flexible linker, were comparable. Melting temperature for the closed trimers increased from 67.93 °C for v2 to 74.17 °C for v5, along the lines of the increasing numbers of included modifications for stabilization. Cooperativity of the thermal unfolding was highest for the ConCv2 KIKO and ConCv4 KIKO trimers (Figure 2a left and bottom).

Analysis of the particle size distributions by dynamic light scattering (DLS) further revealed that ConCv1 KIKO NFL was largest (cumulant radius: 10.04 nm), while the other three trimers exhibited comparable sizes (cumulant radii: 7.13 nm (v2), 7.06 nm (v4), 7.16 nm (v5)). As expected, the ConC KIKO gp120 monomer had the smallest hydrodynamic radius (4.83 nm) (Figure 2a right and bottom).

In addition, all protein variants were further examined by negative staining electron microscopy (EM) and single particle analysis (Figure 2b). The data quality for the monomer ConC KIKO gp120 and for the open trimer ConCv1 KIKO NFL were rather poor, as expected, and did not qualify for further analysis by 2-D class averaging. The SOSIP-based trimers ConCv2 KIKO, ConCv4 KIKO, and ConCv5 KIKO revealed an increasing degree of homogeneity, displaying particles with the classical top view windmill structure (Figure S1). This was confirmed by 2-D class averaging of the negative stain data, which showed that the majority of particles were in the native conformation and that, among these, the proportion of particles in the preferred closed conformation continuously increased from 56% for ConCv2 KIKO to 87% for ConCv4 KIKO and 94% for ConCv5 KIKO (Figure 2b).

### 3.3. Binding Profiles of bnAbs and Non-nAbs Support Differential Protein Characteristics Introduced by Protein Design

**Figure 3:**
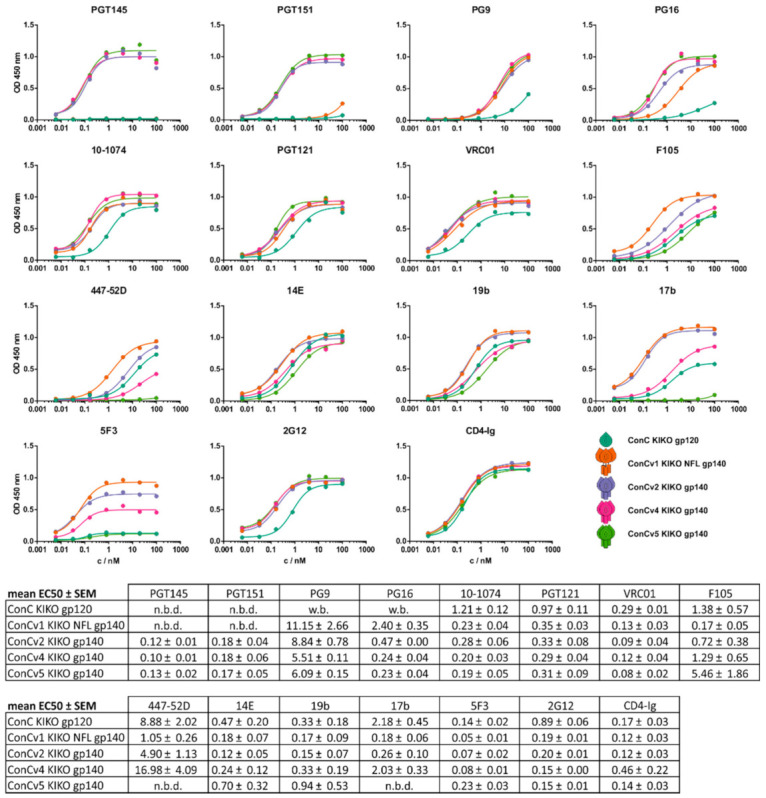
Stabilized ConC trimers show favorable binding profiles to bnAbs and non-nAbs. ELISA binding curves for selected broadly neutralizing (bnAb) and non-neutralizing (non-nAb) antibodies. Teal: ConC KIKO gp120; orange: ConCv1 KIKO NFL; lilac: ConCv2 KIKO; pink: ConCv4 KIKO; green: ConCv5 KIKO. PGT145 (trimer specific); PGT151 (gp120/gp41-interface and fusion peptide targeting); PG9 and PG16 (V2 apex targeting and quaternary structure dependent); PGT121 and 10-1074 (V3 supersite); VRC01 (CD4bs); F105 (malfolded CD4bs); 19b, 14E, and 447-52D (V3 loop); and 17b (co-receptor binding site, CD4 induced state), 5F3 (MPER), and 2G12 (glycan-dependent). Curves are shown for one representative of two experiments. Mean EC50 values ± SEM are given. n.b.d.: no binding detected; w.b.: weak binding.

Binding characteristics of bnAbs and non-nAbs facilitate insight into some of the structural features of Env. Hence, we performed Ni-NTA capture ELISA experiments with a comprehensive panel of bnAbs and non-nAbs, targeting all relevant sites in the Env protein (Figure 3). To ensure comparability of data generated for the gp120 monomer and the gp140 trimers, we determined the optimal coating amount for gp120 as the triple molar amount of gp140 (data not shown). This was further confirmed by the binding data for CD4-Ig.

Antibodies PGT145 (trimer specific) and PGT151 (gp120/gp41-interface and fusion peptide targeting) bound the SOSIP-based trimers v2, v4, and v5 with comparable affinities but not the gp120 monomer and ConCv1 KIKO NFL, again suggesting that the gp120 and gp41 subunits in v1 were only loosely attached to gp41 instead of constituting a closed trimer. The two V2 apex-targeting and quaternary structure-dependent antibodies PG9 and PG16 exhibited similar binding patterns to PGT145, but with moderate binding to v1, potentially reflecting their reduced dependence on trimer quaternary conformation compared to PGT145 [44,45]. Binding patterns of bnAbs PGT121 and 10-1074 (V3 supersite) were comparable, with similar affinities for all gp140 trimers and reduced affinity (factor 3–5) for the gp120 monomer. Furthermore, similar results were found for VRC01 (CD4bs). Affinity of non-nAb F105 (malfolded CD4bs) for the four trimers decreased with increasing degree of rigidification. Interestingly, comparable results with even higher divergence and complete absence of binding for v5 were found for antibody 17b (co-receptor binding site, CD4 induced state). Of note, not only were binding affinities reduced but also maximal signal intensities declined with an increasing degree of protein rigidity, suggesting that only a subfraction of the protein was bound by the antibody. Similar binding characteristics (reduction in affinity in part with reduction in maximal signal intensity) were observed for a set of antibodies targeting the V3 loop (19b, 14E, 447-52D), whose exposure should be inhibited by T316W [35]. Non-nAb 5F3 (MPER) bound to all trimers with comparable affinities but declining maximum signals, suggesting that accessibility to this unfavorable epitope could be continuously decreased. Notably, signals for v5 were comparable to those for gp120, which lacks the 5F3 epitope due to truncation of gp41. Binding affinities for 2G12 (glycan-dependent) were almost identical for all gp140 trimers and slightly reduced for the gp120 monomer. Overall, with increasing degree of rigidification of the trimers, we observed a reduced binding of non-nAbs and increased or retained binding of bnAbs (Figure 3).

### 3.4. Quality and Quantity of Induced IgG Responses Is Dependent on the Degree of Immunogen Stabilization

**Figure 4:**
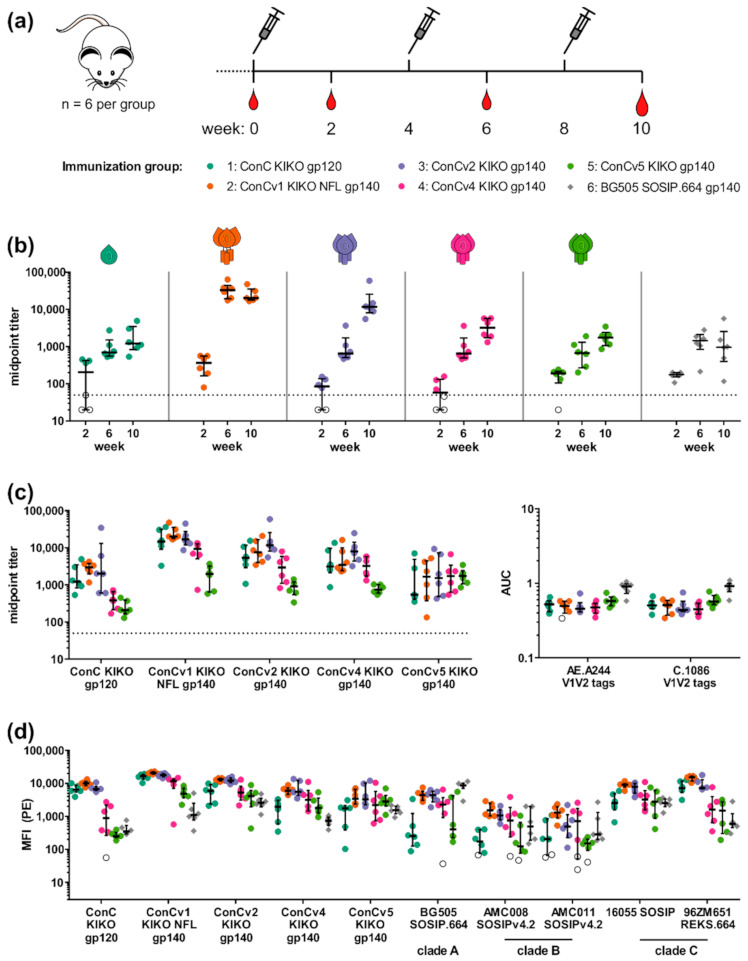
The degree of stabilization of the immunogen impacts quality and quantity of immune responses. (**a**) Immunization schedule of mouse study (n = 6 per group). Immunizations (syringe) and bleeds (blood drop) are indicated at the respective weeks. List of immunization groups is given below. (**b**) Development of autologous midpoint titers over time. Midpoint titers for each animal to the respective homologous protein are given for the three bleeds in weeks 2, 6, and 10. Non-responders (i.e., sera with midpoint titers below the lowest serum dilution factor of 50 represented by the dotted line) are indicated with empty symbols. Data are shown as median with interquartile range. (**c**) Analysis of binding for week 10 sera from groups 1–5 to the autologous and the respective heterologous ConC derivatives. Data are shown as median and interquartile range. The dotted line indicates the dilution factor (50) of the lowest serum dilution (left). Analysis of serum binding to V1V2 proteins (right). Cumulative biding (area under curve, AUC) was determined for the binding curves to the V1V2 proteins and data are shown as median and interquartile range and non-responders are indicated as empty symbols. (**d**) Analysis of IgG binding to all ConC Env variants and a panel of heterologous trimers from clades A (BG505 SOSIP.664), B (AMC008 SOSIPv4.2, AMC011 SOSIPv4.2), and C (16055 SOSIP, 96ZM651 REKS.664) in a Luminex multiplex assay. Non-responders were determined based on the binding of sera from naïve animals to the respective Env variants. Data are shown as median and interquartile range, and non-responders are shown as empty symbols.

We next asked whether the differences in protein characteristics and antigenicity patterns of the five analyzed ConC variants would translate into differential immunological outcomes. Therefore, Balb/c mice (n = 6 per group) were immunized three times (weeks 0, 4 and 8) with 20 µg of protein adjuvanted with liposomal MPLA, a Toll-like receptor 4 agonist (Figure 4a). Animals were bled at the preimmune timepoint and 2 weeks after each immunization (weeks 0, 2, 6, and 10). In addition to groups 1–5 that each received one the five ConC derived variants, an additional group was immunized with BG505 SOSIP.664 trimers (group 6). The degree of modification of the immunogen administered to this reference group was best comparable to ConCv4 KIKO.

We longitudinally analyzed binding antibody responses only to the respective autologous proteins, due to volume limitations of sera from the interim bleeds (Figure 4b). Development of midpoint titers over time differed depending of the degree of stabilization. While titers plateaued after the second immunization for animals immunized with the more open trimer ConCv1 KIKO NFL, titers for animals immunized with the more closed trimers ConCv2 KIKO, ConCv4 KIKO, and ConCv5 KIKO increased with each immunization. Of note, maximum titers inversely correlated with the degree of stabilization of the trimers, suggesting that the display of immunodominant epitopes to B cells and thus the stimulation of antibody responses directed to immunodominant decoy epitopes could be avoided. Titers for animals immunized with the gp120 monomer ConC KIKO were overall lowest amongst the ConC variants (median titer w10: 1215) and were scarcely boosted by the third immunization (fold increase from w6: 1.7). Midpoint titers for group 6 (BG505 SOSIP.664; median titer w10: 970) reached similar levels to those of group 5 (ConCv5 KIKO; median titer w10: 1739). However, the temporal kinetics with minimal to no boosting effect of the third immunization in group 6 (BG505 SOSIP; fold increase from w6: 0.7) resembled more that of group 2 (ConCv1 KIKO NFL; fold increase from w6: 0.6) than that of group 5 (ConCv5 KIKO; fold increase from w6: 2.6).

The hypothesis that immunization with the more open immunogens mainly induced reactivities towards open structures was further supported when we analyzed binding of the week 10 sera from animals immunized with different ConC variants (groups 1–5) towards all of the respective other ConC variants (Figure 4c left). Midpoint titers of sera from animals immunized with the completely closed trimer ConCv5 KIKO (green) were comparable for all tested readout trimers (median titers ranging from 1528 to 1739), independent of their degree of stabilization, and—with the exception of two high responders—lowest for the gp120 monomer (median titer 541). These findings indicate that the induced antibodies most probably were not directed against immunodominant decoy epitopes accessible especially on open trimers (or the monomer) such as the interface region, but were rather directed against sites presented on the surface of the closed trimer structure available on all readout proteins. In contrast, midpoint titers in sera from animals immunized with the more open trimer variants ConCv1 KIKO NFL (orange) and ConCv2 KIKO (lilac) were uniformly high with the respective autologous readout protein (median titers of 20,260 and 16,739, respectively) and, compared to the other groups, also with the gp120 monomer (median titers of 2972 and 2014, respectively). Nevertheless, in these sera, titers against the closed trimer ConCv5 KIKO were rather low (median titers of 1671 and 1528, respectively) compared to the autologous titers. Titers for the group of animals immunized with ConCv4 KIKO (pink) showed a similar pattern, however, binding to the gp120 monomer was rather low (median titer: 384) and more comparable to that of animals from the ConCv5 KIKO (green) group (median titer: 210). Interestingly, sera from animals immunized with the ConC KIKO gp120 monomer (teal) exhibited midpoint titers towards the v1, v2, and v4 readout trimers that were comparable with their respective autologous serum reactivities (median titers of 14,765 vs. 20,260; 5342 vs. 11,662; and 3115 vs. 3219, respectively). However, this was less the case for the v5 readout trimer where reactivity was in addition lowest (median titers of 541 vs. 1739). This again suggested a closed structure of ConCv5 KIKO that apparently provided minimal binding surfaces for antibodies induced by open structures.

In order to characterize the responses in more detail, we assessed binding to two V1V2 subunit proteins (AE.A244 V1.V2.tags from CRFO1_AE and C.1086 V1-V2.tags from subtype C) (Figure 4c, right). V1V2 binding antibodies have been described as one of the correlates of protection inferred from the RV144 trial [21] and may therefore be highly desirable. The V3 loop, in contrast, is regarded as a decoy epitope only capable of inducing strain-specific responses. Reactivities towards V3 should therefore be avoided (reviewed by [11]). Overall, reactivity towards the two different V1V2 subunit proteins was comparable, with a slight trend towards higher reactivity of sera from animals immunized with highly stabilized clade C trimer ConCv5 KIKO trimer (group 5) but also BG505 SOSIP.664 (group 6), which, however, did not reach statistical significance.

Next, we asked whether induced antibody responses would be capable of binding trimers from other viral clades. We therefore analyzed Env binding IgG antibody responses in a Luminex multiplex assay towards four closed trimers from clade A (BG505 SOSIP.664), clade B (AMC008 SOSIPv4.2 [35], AMC011 SOSIPv4.2 [46]) and clade C (16055 SOSIP [47]) as well as one uncleaved open trimer from clade C (96ZM651 REKS.664) (Figure 4d). Key findings from the previous ELISA analysis towards the panel of respective heterologous ConC proteins could be confirmed. Sera from all groups bound to the clade A trimer BG505 SOSIP.664. The two clade B trimers AMC008 SOSIPv4.2 and AMC011 SOSIPv4.2 were recognized by most animals but with comparably weak signals. In contrast, the two heterologous clade C trimers were bound by sera from all animals. Binding patterns of all groups towards 16055 SOSIP and 96ZM651 REKS.664 resembled those of ConCv2 KIKO and ConCv1 KIKO NFL, respectively. Interestingly, these two pairs share a similar degree of stabilization.3.5. Breadth and Magnitude of Env-Specific IgG Linear Epitope Recognition Is Linked to the Immunogen Conformation

**Figure 5:**
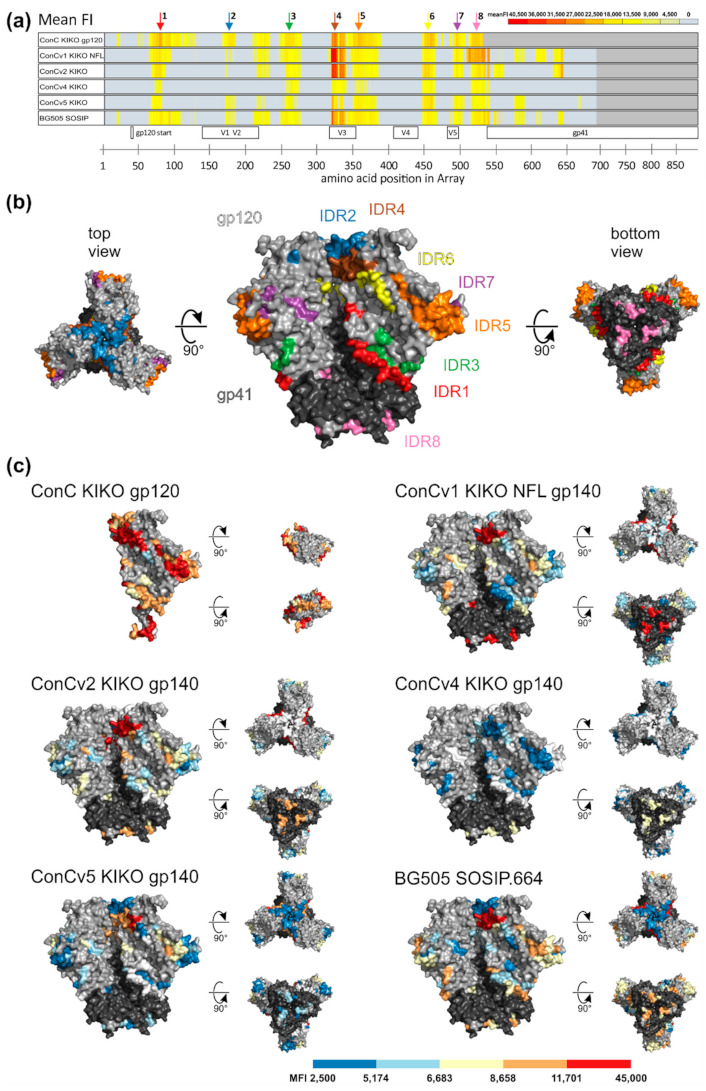
Degree of Clade C consensus immunogen stabilization impacts Env IgG recognition patterns. (**a**) Heat map of mean fluorescence intensity (FI) plotted against individual antigenic regions along the entire HIV-1 Env as included in the peptide microarray. Heat maps of antigenic regions targeted by Env-specific IgG responses are shown for all vaccination groups 2 weeks after the final vaccination. Each row represents one vaccination group comprised of six animals each. FI values corresponding to each 15-mer peptide were mapped to the 10 full-length Env sequences included in the peptide array. Responses above 2500 FI (assay background) after baseline subtraction (pre-vaccination response) were considered positive and the maximum response was selected per position. The mean FI depicted in the graphs was calculated from the maximum FI per amino acid position of each vaccinee per group and is shown for positive responses against peptide positions with a response in at least two animals. Immunodominant regions (IDRs) 1–8 are indicated by colored arrows. Dark grey areas demark the end of the immunogens. (**b**) IDRs are depicted on the accessible surface area (ASA) of a representative pre-fusion Env trimer (PDB: 6CK9; ConC_Base0). (**c**) Mean FI values for each immunization group are mapped on the trimer surface within the previously defined IDRs. For the color code, all positive responses were ranked and distributed in five groups of the same size. Negative responses are shown in white.

In order to elucidate Env IgG binding patterns elicited by different immunogens, vaccine-induced HIV-1 Env-specific recognition was mapped for all Balb/c vaccination groups (n = 6 per group) using microarrays of linear overlapping 15-mer peptides covering the Env protein. The frequency (FOR) and magnitude (mean FI) of the IgG response (2 weeks after the final vaccination) against individual antigenic regions of Env contained in the backbone of the microarray are depicted in Figure 5a and Figure S2a according to immunization group.

We found that the 5 clade C consensus Env variants elicited responses against eight distinct linear immunodominant antigenic regions (IDRs) within the HIV-1 Env (Figure 5a), namely within the C1 (IDR1), V2 (IDR2), C2 (IDR3), V3 (IDR4), C3 (IDR5), C4 (IDR6), and V5-C5 regions (IDR7), and at the gp120-gp41 interface (IDR8). Representative ConC sequences and HXB2 positions of all IDRs with strength of recognition are listed in Figure S2d. All eight IDRs were color coded on the accessible surface area (ASA) of a pre-fusion closed Env trimer (PDB: 6CK9, ConC_Base0 [22]) (Figure 5b). Furthermore, within these IDRs, the mean FI for each individual group was depicted on the Env structure (Figure 5c). In this well-ordered pre-fusion trimer structure, IDR2 (V2, blue) and IDR4 (V3, brown) are located towards the apex of the trimer. IDR 1 (C1, red) and IDRs 5–7 (C3, orange; C4, yellow; V5C5, violet) are contained in the inner and outer domains of gp120, respectively. IDR3 (green) is located in C2 and IDR8 (pink) encompasses the N-terminal gp120 as well as the C-terminal gp41 around the furin cleavage site. Overall, most IDRs are accessible at least partially on the trimer surface. The strongest and most frequent responses for each IDR were elicited in mice vaccinated with the ConC KIKO gp120 monomer, followed by the most open trimer, ConCv1 KIKO NFL. Each step of trimer stabilization diminished the frequency and strength of the antibody targeting of the HIV-1 antigenic regions, which is especially apparent for IDRs 1–5. IDRs 6–8 at the C-terminal end of gp120 are less impacted by the immunogen conformation. Interestingly, the most stabilized trimer ConCv5 KIKO seemed to induce broader Env-responses than ConCv4 KIKO. Further, with diminishing overall immunogenicity, Env-responses between individual mice became more variable, as was seen for ConCv4 KIKO and ConCv5 KIKO variants (data for individual animals not shown). ConCv2 KIKO and BG505 SOSIP.664 show similar recognition patterns of antigenic regions.

### 3.5. Env-Specific-IgG Epitope Variant Recognition Is Linked to Immunogen Conformation and Sequence

Next, we analyzed the impact of immunogen structure on IgG recognition of the various peptide variants included in the array for the 10 IDRs. Figure S2b shows the strength of IgG targeting of the backbone array sequences as well as the additional antigenic variants at 15 previously identified immunodominant regions. The impact of immunogen structure on overall immunogenicity also extended to variant recognition, with a decline in the numbers of variants recognized linked with the increase in trimer stabilization. This became especially apparent for the strongly recognized IDR 4 that includes the tip of the V3 region (Figure S2c). The broadest response of the ConC immunogen variants was observed for the relatively open trimers ConCv1 KIKO NFL and ConCv2 KIKO, whereas the more stabilized trimers of the ConCv4 KIKO and ConCv5 KIKO immunogens elicited much weaker and less broad IgG responses against the V3 region of the HIV-1 Env. Of note, no peptide variant exactly matching the ConCv5 KIKO immunogen sequence with T316W is included in the array. Interestingly, an effect of immunogen sequence on the depth of the Env-specific IgG response could also be observed, as variant recognition differed between mice vaccinated with the clade A-based BG505 SOSIP.664 trimer and the clade C-based ConC immunogens, especially in V3. Here, a clustered recognition of closely related sequences could be observed for the responses elicited by the ConC immunogens but not for BG505 SOSIP.664, which in turn elicited a broad V3 response. (Figure S2c).

## 4. Discussion

Induction of broadly neutralizing antibodies (bnAbs) that confer sterile immunity will probably be a key feature of an effective HIV-1 vaccination strategy. The structure and function of HIV-1 Env has been the center of intense research efforts, being the sole viral protein on the surface of virions. Over the years, increasing the stability of the HIV-1 Env trimer and thereby generating native-like pre-fusion closed trimer mimetics has been deemed extremely important (as reviewed by [18]).

In the current study, we presented a set of Env variants (one gp120 monomer and four gp140 trimers) derived from a novel clade C consensus sequence. These four trimers were stabilized in a stepwise manner by selective incorporation of previously identified amino acid substitutions that help stabilize native-like trimer mimetics. With an increasing degree of stabilization, we observed improved biochemical and biophysical protein properties as well as favorable antigenicity profiles towards bnAbs and non-nAbs. When tested as immunogens in mice, these in vitro findings translated into a favorable immunological outcome.

Biochemical analyses revealed that the predicted stepwise increasing degree of stability introduced by our protein design turned out as intended. The mostly unmodified trimer ConCv1 KIKO NFL, where the gp120 subunit was only loosely attached to gp41 by the native flexible linker [9], tended to aggregate. However, it transformed into the well-behaved trimer ConCv5 KIKO, which is secreted from cells as homogeneous protein species consisting primarily of trimers. Of note, neither of our presented trimers were purified by negative or positive selection. In parallel, manufacturability of the protein variants could be improved, as derived from continuously increasing trimer yields. For ConCv1 KIKO NFL, we repeatedly observed different multimerization states of the protomers under non-reducing denaturing conditions. Interestingly, this finding was not confirmed under native conditions where only trimers could be found. Analytical SEC of final protein pools (after preparative SEC) also did not suggest the presence of multimers of trimers.

Further biophysical analyses with regard to thermal unfolding, structural integrity, and particle size confirmed our initial assessment. The ConCv1 KIKO NFL trimer appeared more open with a cumulant radius of 10 nm. The particle sizes of the three SOSIP-based trimers ConCv2, v4, and v5 were comparable. Melting temperatures, however, continuously increased, albeit not to the same extent as has been reported previously for the clade C 16055 trimer that was stabilized in a comparable manner [34]. Interestingly, the modifications added to v5 (amongst others a second disulfide bond) increased the melting temperature but at the same time led to a loss in cooperativity of the unfolding compared to v2 and v4. Nevertheless, structural integrity gauged by the proportion of closed trimers in negative stain electron micrographs was highest for ConCv5 KIKO and ConCv4 KIKO without enriching closed trimers by either negative or positive selection-based purification strategies.

Continuously decreasing binding affinities and overall signal intensities of several non-nAbs (F105, 447-52D, 17b, 5F3) supported our intended stabilization approach. Along the same lines, quaternary structure-dependent bnAbs (PGT145, PGT151, PG16) primarily bound to the most stabilized trimers ConCv4 KIKO and ConCv5 KIKO. For all other non-structure-dependent bnAbs, we found comparable binding patterns for all trimers, which was not surprising based on their high similarity. Interestingly, binding affinities of some non-structure dependent bnAbs (10-1074, PGT121, 2G12) towards the gp120 monomer decreased by a factor of three to six compared to the trimers. However, overall, binding of antibodies to the gp120 monomer was in most cases intermediate.

Immunization of Balb/c mice confirmed observations from the in vitro assays. The more open trimers induced autologous Env binding titers more rapidly and with higher plateaus. Based on responses towards selected Env regions (V1V2 and V3), however, quantity did not go hand in hand with quality. We therefore hypothesized that the high titers in mice immunized with more open trimers were primarily driven by antibodies targeting more open structure, including V3-directed antibodies (Figure S2b). Almost all mice produced antibodies that bound the most stabilized trimer ConCv5 KIKO. Interestingly, the median midpoint titers towards this protein were nearly identical for all groups except group 1 (immunized with the gp120 monomer). Only the scatter of the midpoint titers induced in the single animals within the individual groups increased with decreasing degree of stabilization.

The reduction in overall immunogenicity with increasing degree of stabilization was confirmed by peptide microarray analysis. While the overall pattern of IgG Env recognition was comparable between the gp120 monomer, the ConC trimers, and also the clade A BG505 SOSIP.664, we observed a decrease in breadth and depth of IgG recognition of linear gp120 epitopes with increasing stabilization of the ConC immunogens. This was most apparent in the more N-terminal region of gp120 (IDR5). Recognition of the linear V3 region (IDR4) continuously decreased with increasing degree of conformational stability and was almost completely abrogated in animals immunized with ConCv5 KIKO. This variant contained the T316W substitution designed to retain the V3 loop in its ground state location, and its capability to abrogate induction of V3 directed antibodies has been demonstrated before in mice and in rabbits [35]. Binding of the V1V2 region, which has been associated with protection in RV144 [27,42], was low towards the more open trimers v1, v2, and v4, but rather strong towards the most stable trimer ConCv5 KIKO and, interestingly, also towards the ConC KIKO gp120 monomer. Responses towards BG505 SOSIP.664 were comparable to ConCv5 KIKO. In contrast to the differential V1V2 recognition found with the 15-mer peptides by peptide microarray analysis, binding assessed by ELISA towards the V1V2 recombinant proteins AE.A244 and C.1086 covering the complete V1V2 region was more homogeneous. More biologically relevant data might be obtained using V1V2 gp70 scaffolds that mimic a more native conformation of this region. While offering the opportunity to scan Env-specific antibody responses against a large variety of peptides, the peptide microarray analysis is limited in that it reports on binding of linear 15-mer epitopes. Taking this into consideration, however, we nevertheless confirmed our previous finding of an inverse correlation between the degree of conformational stability and overall immunogenicity of our ConC-derived immunogens in mice.

Despite the limited antibody repertoire of mice, this observation is also in line with earlier peptide microarray analyses of clinical trials with Env-based immunogens conducted by us [38,39,40,43] and others [42,48]. Immunogens based on gp120 monomers such as those used in RV144 [42] and UKHVC003 [38,43] in general induced stronger and broader Env-specific IgG responses, targeting various antigenic regions on Env, while the membrane-tethered, more native-like trimeric proteins encoded by MVA-CMDR used in the HIVIS and TaMoVac trials [38,39,40] induced more focused and narrow antibody responses.

Despite favorable biochemical, biophysical, and—to the extent analyzed—immunological properties of ConCv4 KIKO or ConCv5 KIKO, we refrained from probing neutralizing activity of the obtained mouse sera for several reasons: (i) Volumes of sera were small and did not allow a comprehensive analysis of neutralization potency and breadth. (ii) Pseudotype assays performed with mouse sera following Env immunization are known to suffer from a high background that cannot easily be removed by e.g., negative selection or affinity purification. (iii) Mice have a limited antibody repertoire that does not match the diversity of the human response. It is therefore well accepted that following selection of promising candidates in mouse models, breadth and potency of neutralizing antibodies is generally determined in guinea pigs or rabbits. Immunizations with a single stabilized HIV Env, including some clade C stabilized trimers, have so far proven the potential to induce neutralizing antibodies, which are in most cases limited to the autologous pseudovirus and tier 1 pseudoviruses. Broadly neutralizing nanobodies have successfully been induced in camels. However, some limited breadth of neutralizing antibodies towards tier 2 isolates has been observed only in the context of prime-boost regimens. Exemplarily, using the fusion peptide coupled to a carrier providing T cell help followed by booster immunizations using a carefully selected panel of stabilized Env trimers [49] or using a panel of glycan-modified HIV NFL envelope trimers representing different HIV clades displayed on liposomes [50], proved successful in the induction of tier 2 neutralizing antibodies. Great hopes are also associated with a strategy where a carefully orchestrated series of consecutive guiding immunogens are administered to drive maturation of initially targeted naïve B cell lineages towards bnAb responses [51].

The experiments and results presented in this study were generated within in the European HIV Vaccine Alliance (EHVA), one of the two major HIV vaccine networks funded under the EU H2020 program. The ConCv5 KIKO Env variant studied here is under further investigation in rabbit and NHP trials. These and other animal trials explore the potential of this stabilized trimer and rationally designed derivatives thereof to contribute to the induction of neutralization breadth, pursuing some of the strategies described above [52]. The results of the here presented study ultimately contributed—together with additional data—to the selection and promotion of the described ConCv5 KIKO pre-fusion stabilized clade C trimer for GMP manufacturing and testing in a phase 1 clinical trial.

## 5. Conclusions

Stepwise stabilization of a clade C consensus based (ConC) Env immunogen resulted in optimized biochemical and biophysical protein traits such as production yields, thermostability, particle size as well as overall structure and antigenicity. While immunization of Balb/c mice both with the monomeric ConC gp120 and the open trimer mimetics yielded Env specific IgG immune responses towards unfavorable and in part immunodominant epitopes, the closed trimers triggered antibodies mainly targeting conserved sites exposed on the surface of closed trimer structures. Whilst ConCv5 KIKO and derivatives thereof are currently being analyzed in rabbits and non-human primates in more detail, GMP manufacturing of two ConCv5 components has been initiated to prepare for a phase 1 clinical trial.

## Data Availability

Not applicable.

## Supplementary Materials

The following are available online at https://www.mdpi.com/article/10.3390/vaccines9070750/s1, Figure S1: Representative sections of micrographs from negative staining electron microscopy, Figure S2: Immunogen conformation and sequence influence depth and breadth of IgG response to HIV-1 Env antigenic regions.
