# Supplementary material for: Stepwise Conformational Stabilization of a HIV-1 Clade C Consensus Envelope Trimer Immunogen Impacts the Profile of Vaccine-Induced Antibody Responses
Source: Vaccines (Basel). 2021 Jul 6;9(7):750. doi: 10.3390/vaccines9070750 (PMC8310183; doi:10.3390/vaccines9070750)
Supplement: Supplementary file 1 [file vaccines-09-00750-s001.zip › vaccines-1224229-supplementary.pdf]

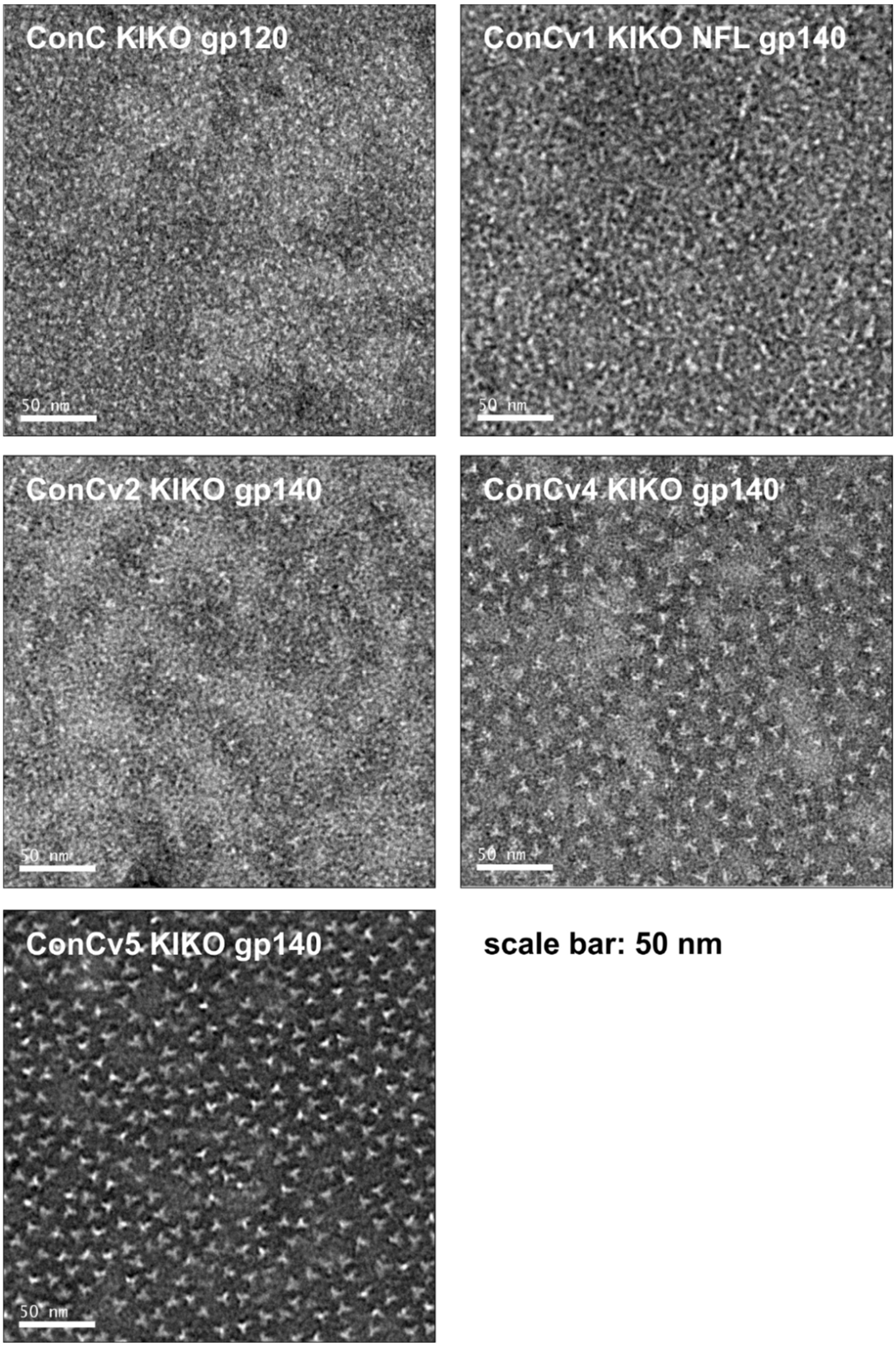

Figure S1. Representative sections of micrographs from negative staining electron microscopy. Shown are image details of micrographs acquired by negative staining electron microscopy. Scale bar represents 50 nm. Names of individual Env variants are given.

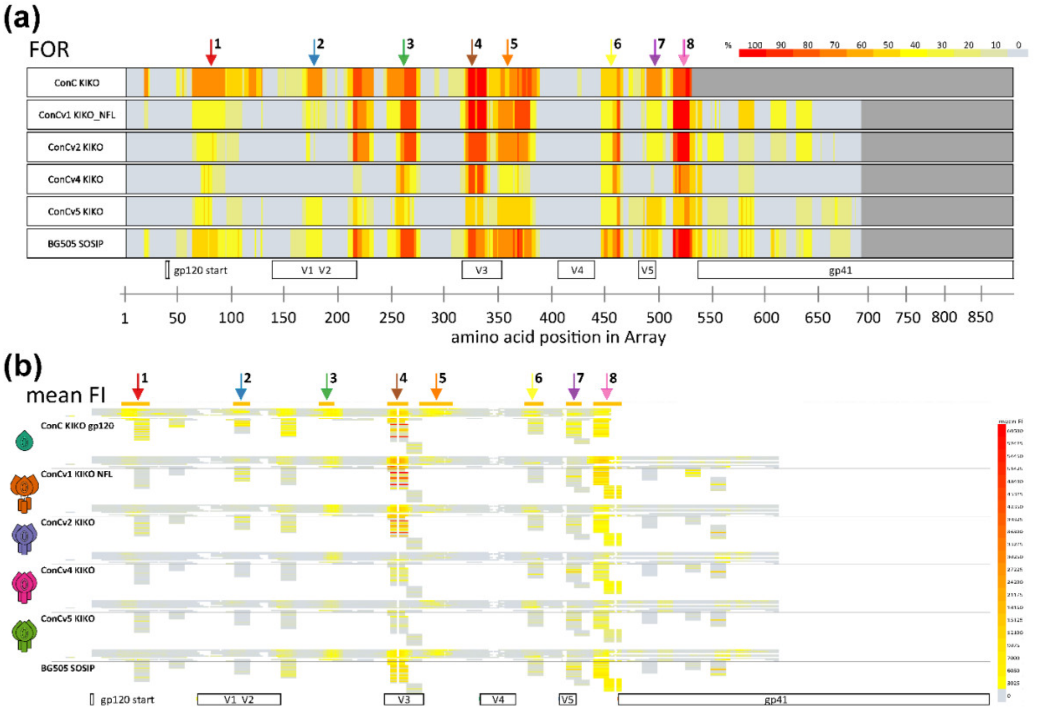

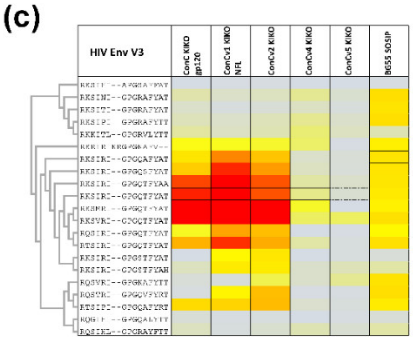

(d)

IDR#	Array position	HXB2 position	Env region	representative sequence (ConC)
IDR_1	67-93	62-88	C1	EKEVHNVWATHACVPTDPNPQEMVLEN
IDR_2	173-188	163-178	V2	TEIRDKKKKEYALFYR
IDR_3	254-268	236-250	C2	TGPCNNVSTVQCTHG
IDR_4	319-338	301-320	V3	KNTRKSIRI--GPGQTFYAT
IDR_5	349-380	330-360	C3	HCNISEEKWNKTLQRVSKKLKEHF-PNKTIKF
IDR_6	449-466	430-447	C4	VGRAMYAPPIAGNITCKS
IDR_7	488-502	465-479	V5C5	TETFRPGGGDMRDNW
IDR_8	514-540	491-515	gp120-gp41	IKPLGIAPTKAKRRVVEREK--RAVGI

Figure S2. Immunogen conformation and sequence influence depth and breadth of IgG response to HIV-1 Env antigenic regions. (**a**) Heat map of frequency of responders (FOR) plotted against individual antigenic regions along the entire HIV-1 Env as included in the peptide microarray. Heat maps of antigenic regions targeted by Env-specific IgG responses are shown for all vaccination groups two weeks after the final vaccination. Each row represents one vaccination group comprised of 6 animals each. Responses above 2500 FI (assay background) after baseline subtraction (pre-vaccination response) were considered positive and the maximum response was selected per position. The frequency of positive responses (>2500 FI) was calculated per amino acid position and is given as frequency of responders (FOR). Immunodominant regions (IDRs) 1–8 are indicated by colored arrows. Dark grey areas demark the end of the immunogens. (**b**) Heat maps of antigenic regions targeted by Env-specific IgG responses are shown for all vaccination groups two weeks after the final vaccination. Each block represents one vaccination group comprised of 6 animals each. Each row represents the mean FI values per vaccination group corresponding to each amino acid mapped to the 10 full-length Env sequences included in the peptide array (above the black line) as well as all additional sequences at areas of special interest included in the array (rows below the black line). Responses above 2500 FI (assay background) after baseline subtraction were considered positive and the mean response for each position was calculated per vaccination group. Data for the length of the immunogen is shown per group. Immunodominant regions (IDRs) 1–8 are indicated by orange lines. (**c**) Heat map of signal intensities for each peptide variant included for HIV-1 Env V3 (IDR_4) and vaccination group. The mean FI for each variant is shown per vaccination group for all HXB2 aa304-320 peptides included in the array. Sequences are sorted by phylogenetic relationship using Fast Tree Full sorting (CLUSTAL 2.1 Multiple Sequence Alignments). Immunogen sequences included in the peptide microarray are boxed for each vaccination group, with the native sequence (T316) boxed for ConCv5 KIKO. (**d**) Summary of immunodominant antigenic regions (IDR). IDRs were defined as regions within the Env covered by 15mer peptides being recognized by at least 60% of mice per vaccination group and with a mean FI for individual amino acids within the region of >10512.17 (mean of all responses in all groups + 2xSD) occurring in one of the vaccination groups.
